# The formation of (NiFe)S_2_ pyrite mesocrystals as efficient pre-catalysts for water oxidation†
†Electronic supplementary information (ESI) available. See DOI: 10.1039/c7sc05452a


**DOI:** 10.1039/c7sc05452a

**Published:** 2018-02-01

**Authors:** Bing Ni, Ting He, Jia-ou Wang, Simin Zhang, Chen Ouyang, Yong Long, Jing Zhuang, Xun Wang

**Affiliations:** a Key Lab of Organic Optoelectronics and Molecular Engineering , Department of Chemistry , Tsinghua University , Beijing , 100084 , China . Email: wangxun@mail.tsinghua.edu.cn; b Beijing Synchrotron Radiation Facility , Institute of High Energy Physics , Chinese Academy of Sciences , Beijing 100049 , China

## Abstract

Designing intricate structures and searching for functional materials has attracted wide interest in nanoscience. Herein we have fabricated (NiFe)S_2_ pyrite mesocrystals in the form of nearly-single crystalline porous cubes, and studied their self-optimization to realize efficient activity toward water oxidation under electrochemical conditions. The growth mechanism of the mesocrystals was a non-classical mechanism, which was initiated by the formation of a large quantity of small nickel sulfide clusters, followed by the aggregation and transformation of these small clusters in an oriented manner. When these mesocrystals were tested for water oxidation under electrocatalytic conditions, the materials served as pre-catalysts and immediately self-optimized to form amorphous S-doped metal (oxy)hydroxides, which are the real catalytically active materials. As a result, the observed overpotential to reach a current density of 10 mA cm^–2^ on glassy carbon electrodes was less than 260 mV. The growth mechanism studied here may provide opportunities for constructing intricate sulfide structures, and the self-optimization process during water oxidation can inspire new thoughts on electrocatalysis.

## Abstract 2

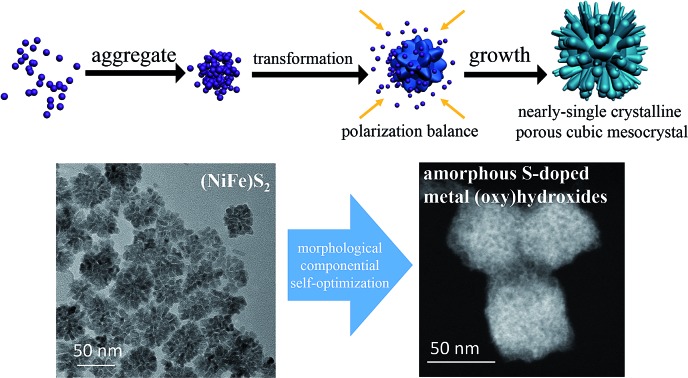
We have fabricated (NiFe)S_2_ pyrite mesocrystals in the form of nearly-single crystalline porous cubes that self-optimize for efficient catalysis of water oxidation under electrochemical conditions.

## Introduction

Many natural minerals exhibit amazingly complex hierarchical morphologies, for example, nacreous layers of seashells, sea urchin spines, egg-shells and corals.^1^,^2^ The above mentioned structured materials are often single or nearly-single crystalline. To describe this kind of intricate structure, the term “mesocrystal” was introduced, which is defined as a mesoscopically structured crystalline material with a common crystallographic orientation.^3^ A mesocrystal is usually the result of non-classical crystallization which involves nano or amorphous building blocks instead of atom/ion/molecule building blocks.^4^ Exploring their growth mechanism could shed light on the rational design of intricate nanostructures. In this context, different strategies to align the building blocks of mesocrystals have been proposed,^4^,^5^ for example, organic matrix, physical field or interparticle force assisted alignment, spatial constraints, mineral bridges, oriented attachments, topotactic reactions, *etc.* Accordingly, some artificial mesocrystals have been designed, like TiO_2_,^6^,^7^ Co_3_O_4_,^8^ ZnO,^9^ polyoxometalate (POM),^10^*etc.* Due to their intricate structures, they are not only promising in mechanical applications,^11^ but also efficient in applications^12^ like lithium storage and photocatalysis.

Natural minerals not only display novel structures, but also provide a large material library for designing functional materials. Pyrites are common minerals that widely exist in metamorphosed ores and metamorphic rocks.^13^ They have been shown to be promising in the design of efficient energy conversion devices^14^ like electrocatalysts^15^ and photovoltaic devices.^16^ Among various different electrocatalytic processes, the oxygen evolution reaction (OER) is one of the bottlenecks in the so-called water cycle, which aims to create an efficient and green energy landscape.^17^ Some pyrite materials, like NiS_2_,^18^ NiSe_2_ 19 and CoS_2_,^20^ have been found to be active in the OER. However, metal pyrites should be easily oxidized under OER conditions according to thermodynamics, and some studies have suggested that the derived metal (hydr)oxides are the main reason for the activity of metal dichalcogenides.^21^–^24^ There are still many unexplored questions in identifying the real active materials of pyrites as OER catalysts. A catalyst should be both a reactant and a product of the reaction according to the International Union of Pure and Applied Chemistry Gold Book. Thus, a material which greatly changes before and after the reaction should not be directly called a catalyst. The structural and componential changes to form catalytically active materials can be seen as a self-optimization process,^25^ and such materials could be called pre-catalysts.^21^

Herein we have constructed (NiFe)S_2_ pyrite mesocrystals in the form of nearly-single crystalline porous cubes (PCs), and used the NiS_2_ pyrite as an example to reveal the growth mechanism. The formation mechanism in this case was a non-classical one that was initiated by the formation of a large quantity of small nickel sulfide clusters, which was then followed by the aggregation and transformation of these small clusters in an oriented manner. As for the OER performance, the Fe-doped PCs showed an overpotential (*η*) of less than 260 mV to reach a current density of 10 mA cm^–2^ (*η*_10_) on glassy carbon electrodes (GCEs). Further characterization techniques like transmission electron microscopy (TEM), soft X-ray adsorption spectroscopy (sXAS) and X-ray photoelectron spectroscopy (XPS) revealed that the pyrites would self-optimize to form amorphous S-doped metal (oxy)hydroxides immediately after several cycles of cyclic voltammetry (CV) scans, which were conducted prior to their performance tests. Accordingly, the Fe-doped PCs, which are actually pre-catalysts, are efficient in both self-optimization and OER performance. We then further confirmed the beneficial role of the doped S with the help of Fe doping in the materials, by further adding S^2–^ into the electrolyte and comparing the sXAS spectra of the amorphous (oxy)hydroxide with or without S doping.

## Results and discussion

### The structure and the growth mechanism of the mesocrystal

**Fig. 1:**
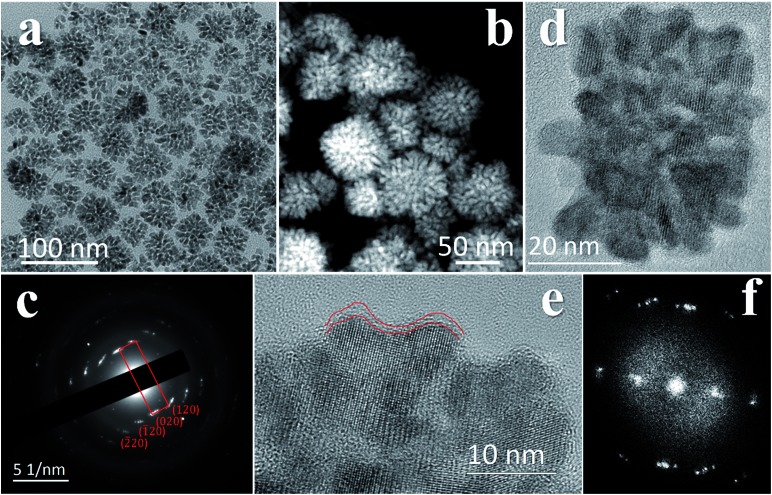
TEM and SAED characterizations of the PC-1 structure. (a) TEM image at low magnification. (b) STEM image. (c) A typical SAED pattern of a single PC particle. The patterns correspond well with that of the NiS_2_ pyrite crystal. (d) A typical HRTEM image of a single PC particle. (e) HRTEM image at higher magnification. The area between the red lines showcases the amorphous shell at the periphery of the particle. (f) The FFT pattern of the entire particle in (d).

The mesocrystal feature of the synthesized PC was confirmed using TEM and selected area electron diffraction (SAED). Fig. 1a shows a typical TEM image of the constructed NiS_2_ pyrite mesocrystals (denoted PC-1). The PC structure resembles sea urchins and is composed of rod-like building blocks (RBBs), however, the entire structure here is cubic rather than spherical like that of urchins. The size of the PC is about 40–50 nm, and some of the particles are tilted on the TEM grid. The scanning TEM (STEM) image (Fig. 1b) clearly depicts the porous feature which is a typical (although not prerequisite) characteristic of the so-called mesocrystals.^4^,^5^ To confirm the mesocrystal structure, high resolution TEM (HRTEM) and SAED were used to detect whether there is a common crystallographic orientation between different RBBs. The HRTEM image suggests high crystallinity of the particles (Fig. 1d). Fig. 1f is the fast Fourier transformation (FFT) image of the entire particle shown in Fig. 1d, and the result shows only a slight orientational distortion of the patterns. The enlarged HRTEM image (Fig. 1e) shows that there are amorphous shells around the PC (for example, the area between the red lines). Element mapping results confirmed the even distribution of metal and S, and very few O signals can be detected (Fig. S1†). Furthermore, a typical SAED pattern (Fig. 1c) of a single PC particle also showed high ordering,^4^,^26^ suggesting a common crystallographic orientation. These results confirmed the mesocrystal features. Overall, the FFT patterns and SAED patterns are consistent with the X-ray diffraction patterns (XRD, Fig. S2†), and all of them correspond well with that of the pyrite NiS_2_ crystal.

**Fig. 2:**
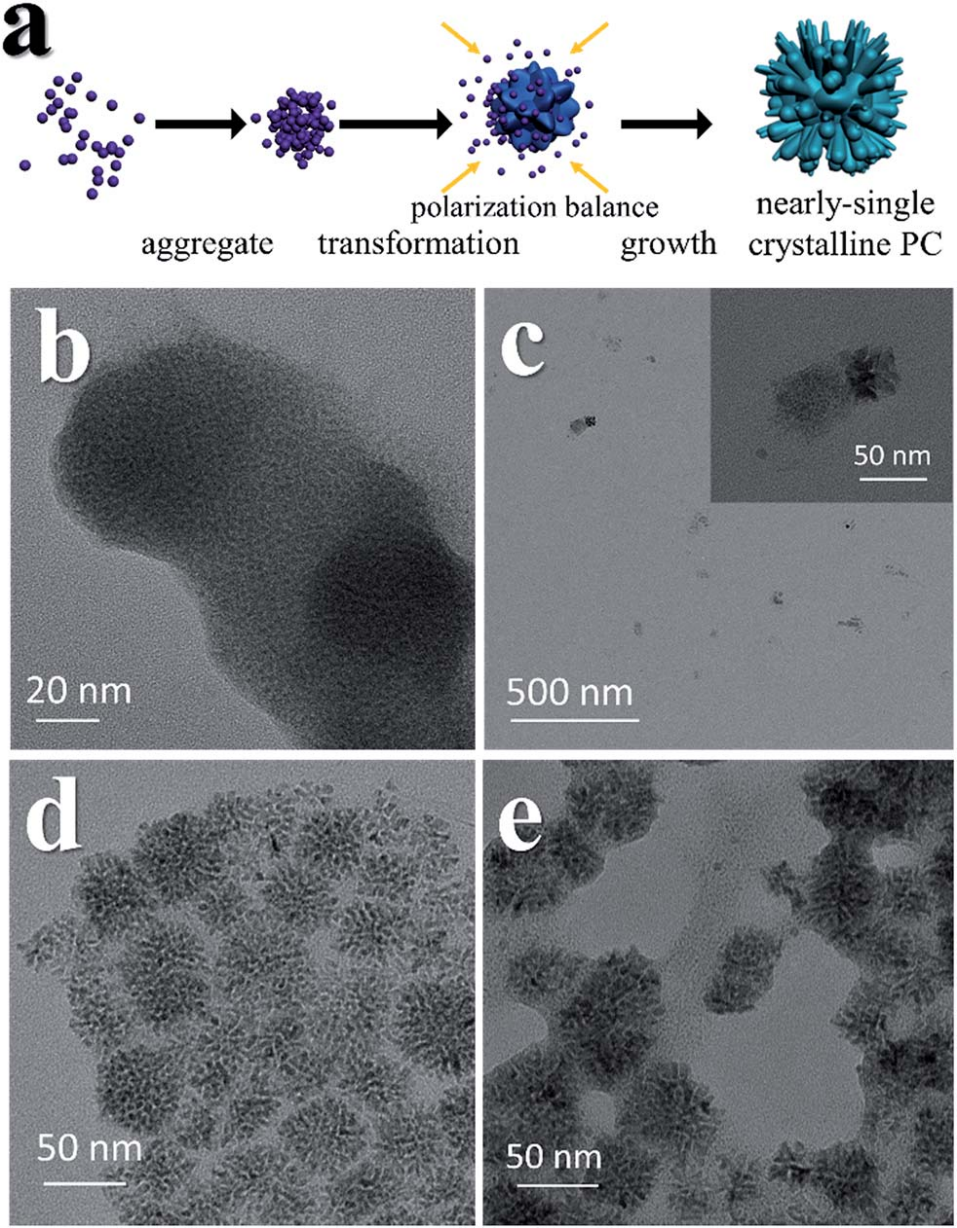
Growth mechanism of the PC structure. (a) A cartoon image illustrating the morphology evolution. (b) Small clusters can be obtained immediately after adding TAA/OAm solution into Ni(NO_3_)_2_/OAm. (c) Products obtained after increasing the temperature to 180 °C. (d) Products obtained after keeping the reaction temperature at 180 °C for 30 min. (e) Products obtained with a reaction temperature of 140 °C for 4 h.

PC-1 was synthesized by mixing Ni(NO_3_)_2_ and thioacetamide (TAA) in oleylamine (OAm), which was followed by heating at 180 °C (see experimental details in the ESI†). TAA can release S^2–^ in OAm. Thus, a large quantity of black precipitate was formed immediately after adding the TAA/OAm solution into the Ni(NO_3_)_2_/OAm solution. The black precipitate was washed and analyzed. Fig. 2b is the corresponding TEM image, which shows that the black precipitate consists of small clusters of less than 2 nm embedded in a gel (Fig. S3†). The formation of nickel sulfide is favored from the viewpoint of the precipitation equilibrium (see below), since the solubility products of nickel sulfides are much smaller than the equilibrium constant of the decomposition of the ammonia complex (we used the data of ammonia to showcase the stability of the ammonia complex). Thus, the concentrations of free Ni^2+^ and S^2–^ in the system were extremely low, making the classical growth mechanism unrealistic here. When the reaction temperature reached 180 °C, the aggregation of small clusters and PC structures could be easily observed (Fig. 2c). When the reaction time was elongated to 30 min (Fig. 2d), PC structures with sizes of about 40–50 nm were the main products. When the reaction temperature was decreased to 140 °C (Fig. 2e), the PC structures were not well defined, and the addition of small clusters onto the PC particles can be clearly seen.
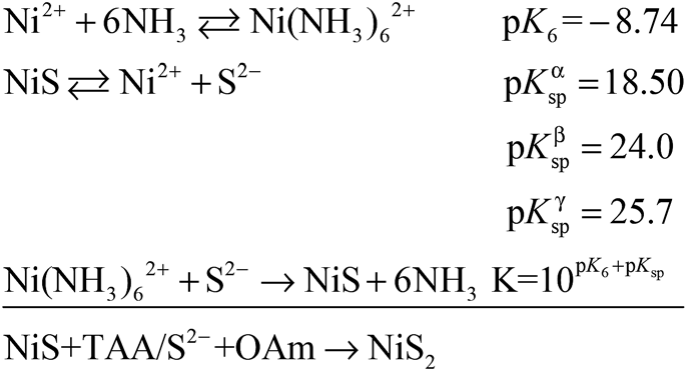


The growth mechanism is illustrated in Fig. 2a. At the beginning, a large amount of small clusters was formed. Then the clusters aggregated and transformed into pyrites. During the process, the crystalline nucleus attracted clusters and restructured them after attachment. This was supported by the amorphous shell around the PC (Fig. 1e and Fig. 2e). The orientations of different RBBs were aligned as the crystal grew. The reason for alignment may be attributed to the polarization of the pyrite crystals.^27^ Once a cluster crystalized onto the nucleus, the polarization within the structure accumulated in an coherent fashion. Consequently, the Hamaker constant and relevant surface energy would increase, leading to the directional attraction of another cluster.^5^,^28^ Theoretical calculations have suggested that the screened electrostatic potential that is generated by an anisotropically charged object is anisotropic at any distance,^29^ but especially at short distance.^27^ Such a potential can be used as the driving force to realize mutual alignment of the RBBs,^27^,^30^ and the appearance of the entire particle should be determined by the polarization of the inherent lattice structure of the crystal when proper reacting conditions are provided. The crystal structure of a pyrite features a space group of *Pa*3, which is a face-centered cubic structure. Because the entire particle would thermodynamically favor small or balanced polarization, forming a cubic structure as the final morphology is thermodynamically favored. This result is also consistent with the fact that the symmetry of a mesocrystal is very often higher than that of its building blocks.^3^,^30^ The reason that the cubic structure remained porous might be due to the proper stabilization of the RBBs. Too strong or too weak stabilization or interaction between them would result in repulsion or immutable collision of the RBBs, leading to different morphologies. The stabilization can be described by the Derjaguin–Landau–Verwey–Overbeek (DLVO) potential. At high temperature, the thermal energy (*kT*, where *k* is the Boltzmann constant) is able to populate all possible states, finding the secondary minimum of the DLVO potential and leading to well-defined porous structures composed of oriented RBBs.^5^ When the temperature is not high enough, the added clusters may collide onto the formed particles and cannot easily adjust their orientations. This effect also explains the ill-defined structures obtained at low temperature (Fig. 2e). On the other hand, high temperature is helpful in providing enough energy to find the thermodynamically favored structures, forming cubic structures.

### Self-optimization to deliver efficient OER activity

**Fig. 3:**
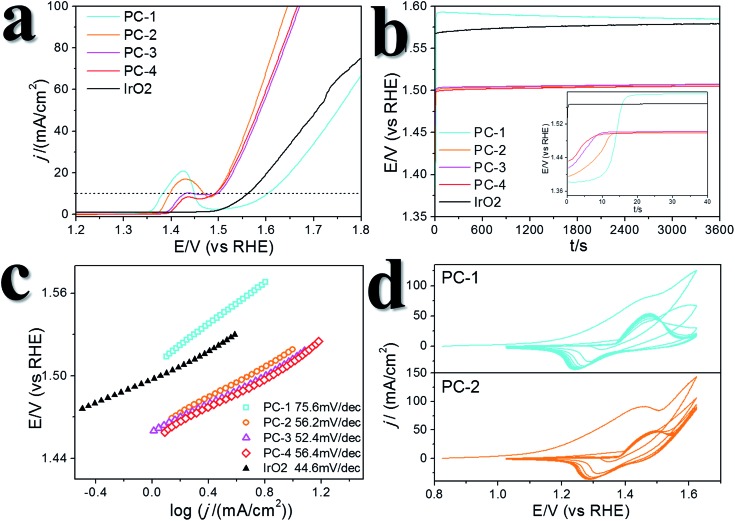
OER performance of the synthesized PC materials. (a) LSV curves. (b) Chronopotentiometry tests (at 10 mA cm^–2^). The inset is the enlarged image of the starting 1 min. (c) Tafel slopes. (d) 10 cycles of CV scans prior to the performance tests.

A certain amount of Fe can be easily doped into the PC structures without changing the morphology (Fig. S4†). (Ni_0.86_Fe_0.14_)S_2_, (Ni_0.74_Fe_0.26_)S_2_ and (Ni_0.63_Fe_0.37_)S_2_ (denoted PC-2, PC-3 and PC-4, respectively) can be obtained by adding the required amount of Fe(NO_3_)_3_ in the synthesis. However, phase separation occurred when a high amount of Fe(NO_3_)_3_ was added (Fig. S4†). XRD patterns demonstrated shrinking of the lattice compared to the pure Ni pyrite, but the pyrite structures were preserved (Fig. S2†). Then, the synthesized PC materials were tested for OER activity in 1 M KOH solutions. PC-1 showed an *η*_10_ of 351 mV (with *iR* correction, where solution resistance was obtained by impedance analysis, Fig. S5,† or 373 mV without *iR* correction). When Fe was doped into the PC, *η*_10_ dramatically decreased to about 252 mV, 256 mV and 248 mV (with *iR* correction, or 264 mV, 269 mV and 262 mV without *iR* correction, Fig. 3a) for PC-2, PC-3 and PC-4, respectively. These values were all much better than that of the benchmark commercial IrO_2_ catalyst. It’s worth mentioning that currently there are two directions in which to explore the OER. One is to design industrially applicable catalyst systems constructed on conducting substrates, and a very large current density^31^ of about several hundred mA cm^–2^ (normalized by the geometry area) and a high concentration of alkaline solution^32^,^33^ are recommended. Another is to investigate the catalytic mechanisms of the OER.^34^,^35^ In the former direction, the substrates could have a profound impact on activity, not only because they can actually provide large real surface areas, but also because of their non-innocent activity or synergistic effects with the catalysts. Directly comparing the *η*_10_ of catalysts on different substrates is not fair. Turnover frequencies (TOFs) might be a more suitable parameter for comparison of catalysts. To study the mechanisms of the OER, it’s better to simplify the system and get rid of the effect of substrates, thus, inert substrates like GCEs are suggested. Here we intend to investigate the mechanism of using pyrites as OER catalysts, therefore GCEs were used. Nonetheless, the observed *η*_10_ are smaller than many reported data (Table S1†).

The Fe-doped PCs showed similar Tafel slopes (Fig. 3c) and electrochemical active surface areas estimated from the double layer capacitances (Fig. S6†).^36^ The Faraday efficiency (FE) was tested using rotating ring-disk electrode techniques (Fig. S7†),^37^ and the results showed high O_2_ production efficiency. The performance stability was tested using both chronopotentiometry (at 10 mA cm^–2^, Fig. 3b) and chronoamperometry tests (at *η* = 300 mV, Fig. S8†), in which the PCs all displayed good stability. The chronopotentiometry tests also confirmed the high FEs of the Fe-doped PCs at around 1.5 V (*vs.* RHE). CV scans were usually carried out prior to the performance tests, however, the effect of CV scans is largely overlooked in some previous OER studies on metal dichalcogenides. We applied 10 cycles of CV scans before other tests (scan rate: 50 mV s^–1^). Typical CV curves are shown in Fig. 3d. The first cycles were all very different to the following cycles. This phenomenon was more obvious when the scan rate was lower (Fig. S9†). After the first cycle of scans, the observed activities were stable in the following cycles. On the other hand, the relationship of the current densities of anodic peaks with the scan speeds demonstrated a faradaic redox process in the bulk but not the surface (Fig. S6†). These results might reflect some kind of structural change or self-optimization of the materials.

**Fig. 4:**
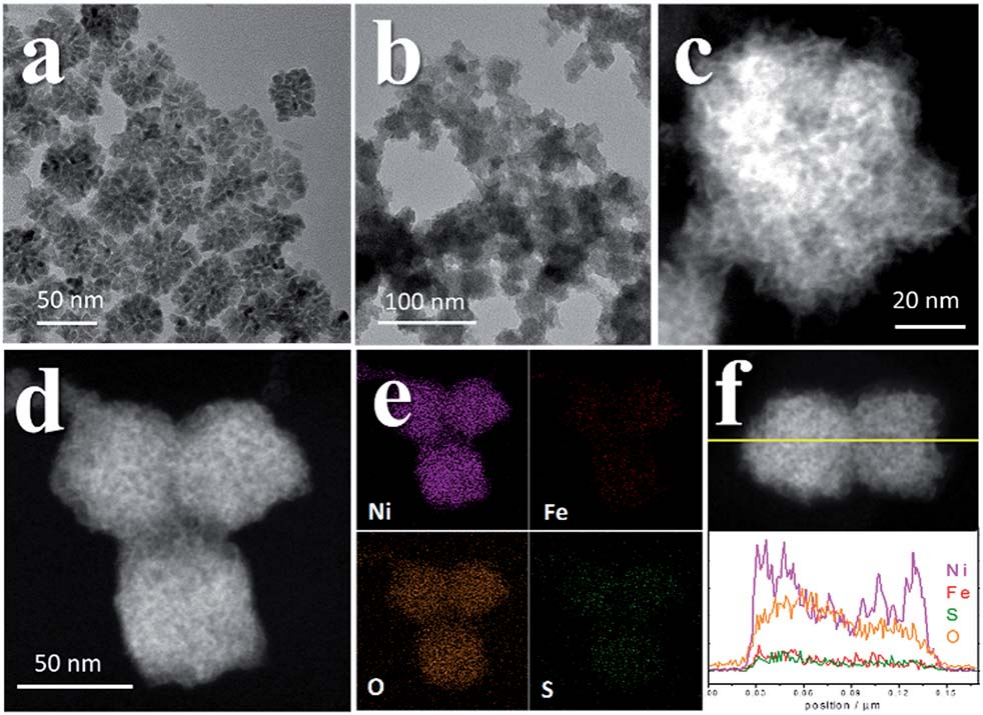
TEM images of PC-2. (a) The original structure of PC-2. (b) TEM images and (c) STEM images of PC-2 after 10 cycles of CV scans. (d, e) Element mapping results and (f) line scanning profile of PC-2 after 10 cycles of CV scans.

We recollected the samples after the CV scans and analyzed them. The structures of all of the pyrite materials had changed a lot. For the pyrites without Fe doping (PC-1), the PC structures were totally destroyed, and irregular particles and nanowires were formed (Fig. S10†). For the Fe-doped PCs, the morphologies evolved into amorphous cubic structures composed of whiskers (Fig. 4, Fig. S11, S12†). Fig. 4a is a typical TEM image of PC-2, and Fig. 4b–f depict the morphology of PC-2 after CV scans. Unlike for the pristine structures, which contain a low amount of O (Fig. S1†), the element mapping and line scanning results confirmed the large amount of O in the structure after CV scans. XPS was also used to study the differences (Fig. S13†). Although the pyrites mainly transformed into metal (oxy)hydroxides, the relative amount of remaining S (compared to the total amount of metal) was higher with a higher content of Fe doping (Fig. S14†). Even after the chronoamperometry tests (at *η* = 300 mV, 1 h), S could not be totally removed, and the relative amount of remaining S was higher with a higher content of Fe doping. However, phase separation occurred with an elevated amount of Fe doping after the stability test (Fig. S15†). According to the XPS results (Fig. S13†), there are two types of S after the CV scans or stability tests: one is the S anion in pyrite structures,^38^ and the other is S–O arising from the surface absorption of oxygen.^20^

**Fig. 5:**
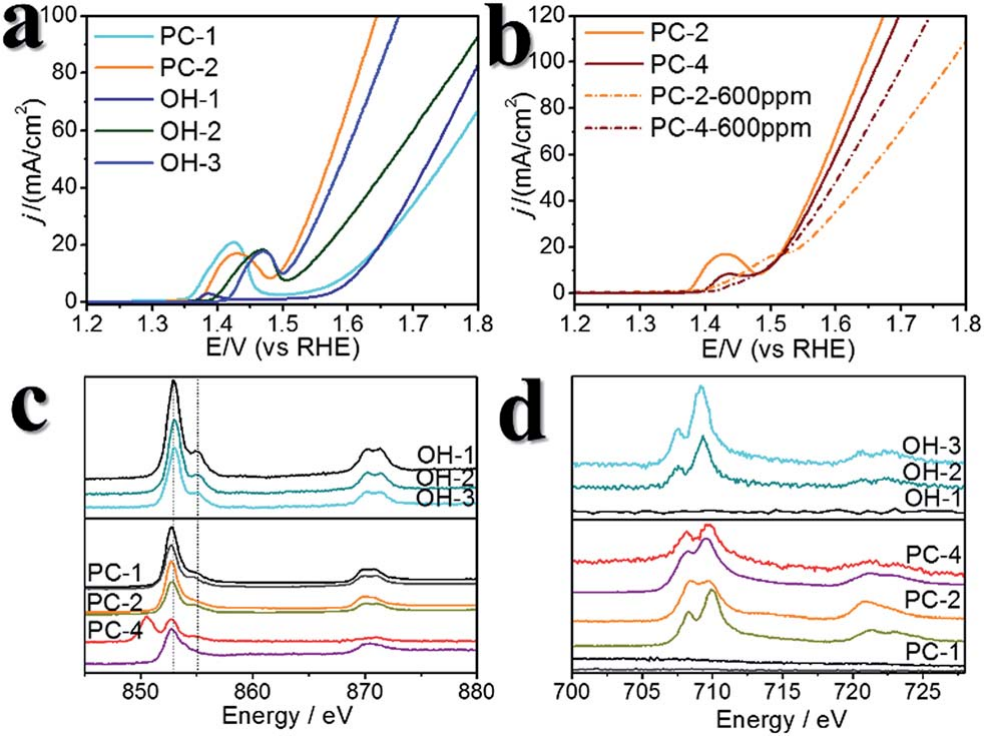
Comparison between pyrite pre-catalysts and (oxy)hydroxides. (a) LSV curves of the (oxy)hydroxides, PC-1 and PC-2. (b) LSV curves of the pyrite pre-catalysts with and without additional S^2–^ in the electrolyte. (c) Ni L-edge sXAS spectra. The upper panel shows the results for the (oxy)hydroxides, and the lower panel shows the results for the pyrite pre-catalysts. In the lower panel, the first spectrum below each label is the data for the corresponding pristine pyrite material, and the second spectrum below each label is the data for the corresponding pyrite pre-catalyst after 10 cycles of CV scans. (d) Fe L-edge sXAS spectra. The labelling manner is the same as that in (c).

To further investigate the influence of S, several controlled experiments were carried out. First, amorphous NiFe (oxy)hydroxides with different Ni : Fe ratios were prepared (pure Ni, Ni : Fe = 6.9 : 1 and Ni : Fe = 3.2 : 1, which were denoted OH-1, OH-2 and OH-3, respectively, Fig. S16†). Using these catalysts for comparison revealed the effect of doped S in pyrites after the CV scans. Their *η*_10_ values were larger than those of the Fe-doped PCs (Fig. 5a, Table S1†), while the TOFs at *η* = 300 mV were much smaller than those of the Fe-doped PCs. OH-1 also demonstrated higher onset potential compared to that of PC-1. When 600 ppm S^2–^ was intentionally added into the electrolyte, the CV curves changed greatly in the case of the (oxy)hydroxides (Fig. S17†). The oxidation peak crossed the reduction peak when the Fe content was high. The results were similar in the case of the Fe-doped PC materials (Fig. S18†). The competitive oxidation of S^2–^ was responsible for the curve changes. On the other hand, the S^2–^ in the electrolyte cannot improve the current density at large *η* values (Fig. 5b). These results suggested that S^2–^ in the electrolyte cannot have a positive impact on OER performance. Then sXAS was used to compare the electronic structures of PC pre-catalysts and (oxy)hydroxides (Fig. 5c, d). The sXAS spectra of the as-synthesized PCs and the PCs after CV scans demonstrated different features compared to those of the (oxy)hydroxides. The high valence metal cations under the OER conditions were deemed to be the active centers. The existence of Fe can assist the formation of high valence Ni under the OER conditions.^39^ Typically, the peaks in the sXAS spectra of higher valence cations were centered at higher energy.^39^ In the case of the PC-2 materials, the spectra became more similar to those of the (oxy)hydroxides after CV scans (Table S2†), with a similar ratio of high valence metals. However, the PC-4 materials showed different features, with lower ratios of high valence metals. But the PC-4 pre-catalysts indeed displayed high activity. Thus, the doped S improves the OER activity of the PC-4 pre-catalysts, and Fe can help to stabilize the S doping. The S and Fe doping may serve to reduce the Gibbs free energy for the formation of high valence states,^39^ or directly influence the reaction pathways. However, we cannot yet be sure from these experiments.

## Conclusions

In summary, we have synthesized (NiFe)S_2_ pyrite mesocrystals and studied their self-optimization in serving as efficient pre-catalysts toward the OER. The features of the mesocrystals were confirmed using TEM and SAED. The formation of this unique structure was due to a non-classical mechanism, which can be depicted by the aggregation and transformation of small nickel sulfide clusters. The growth was modulated by thermodynamics to form a common crystallographic orientation between different RBBs. Since the solubility products of various kinds of metal sulfides are very small, this small cluster based growth mechanism might be applicable in constructing other novel metal sulfide nanostructures. Furthermore, the Fe-doped PC materials were good candidates for the OER. The pyrite materials can self-optimize to form amorphous S-doped metal (oxy)hydroxides, which are the real active materials toward the OER. The doped S helped to enhance the activity of the active materials with the help of Fe doping. The beneficial effect of the doped S here may raise more questions that are interesting to explore: (1) why can S anions survive under highly anodic conditions (stabilized by Fe)? (2) How do the S anions interact with metals after CV scans? (3) Could there be anion type single atom catalysts?

## Conflicts of interest

There are no conflicts to declare.

## Supplementary Material

Supplementary information
