# Supplementary material for: The formation of (NiFe)S2 pyrite mesocrystals as efficient pre-catalysts for water oxidation
Source: Chem Sci. 2018 Feb 1;9(10):2762–7. doi: 10.1039/c7sc05452a (PMC5914147; doi:10.1039/c7sc05452a)
Supplement: Supplementary file 1 [file SC-009-C7SC05452A-s001.pdf]

Electronic Supplementary Material (ESI) for Chemical Science.
This journal is © The Royal Society of Chemistry 2018

The formation of $(NiFe)S_2$ pyrite mesocrystals as efficient pre-catalysts for water oxidation

Bing Ni[a], Ting He[a], Jia-ou Wang[b], Simin Zhang[a], Chen Ouyang[a], Yong Long[a], Jing Zhuang[a] and Xun Wang[a]

[a] Key Lab of Organic Optoelectronics and Molecular Engineering, Department of Chemistry, Tsinghua University, Beijing, 100084, China.
[b] Beijing Synchrotron Radiation Facility, Institute of High Energy Physics, Chinese Academy of Sciences, Beijing 100049, China.

Email: wangxun@mail.tsinghua.edu.cn

Contents:

Experiment details: 3

Supporting Fig.s: 5

Fig. S1. Element mapping and line scanning profile of the PC-1 5

Fig. S2. XRD patterns of the PC structures 5

Fig. S3. STEM images and elemental mapping results of the clusters embedded in a gel. 6

Fig. S4. TEM images of the Fe doped PC structures. 7

Fig. S5. Electrochemical impedance spectra. Frequency range: 0.1Hz to 100k Hz. Potential: 1.53V (vs RHE). .7

Fig. S6. CV scans at different scanning rate to estimate the ECSA and oxidation mechanism (surface vs bulk). 8

Fig. S7. The Faradaic efficiency (FE) was measured based on the RRDE tests. 9

Fig. S8. Chronoamperometry tests. 9

Fig. S9. CV scan at lower scan rate and the corresponding TEM images of PC-2 after the scan. 10

Fig. S10. TEM images of the PC-1 before and after OER tests. (It stability: η=300mV, 1h). 11

Fig. S11. TEM images of the PC-2 before and after OER tests. (It stability: η=300mV, 1h). 12

Fig. S12. TEM images of the PC-4 before and after OER tests. (It stability: η=300mV, 1h). 13

Fig. S13. XPS spectra of the PC materials before and after OER tests. 14

Fig. S14. Composition changes of the PC materials before and after OER tests estimated by the XPS spectra. 15

Fig. S15. Element mapping of the PC-4 after It stability (η=300mV, 1h). 16

Fig. S16. TEM images of the (oxy)hydroxides. 17

Fig. S17. CV curves of the (oxy)hydroxides with or without 600ppm S^{2-} in the electrolyte. 18

Fig. S18. CV curves of the PC materials with 600ppm S^{2-} in the electrolyte. 18

Supplementary tables 19

Table S1. A summary of the OER activity 19

Table S2. The contents of high valence metals. 21

Reference 22

Experiment details:

Chemicals: $Ni(NO_3)_2{\cdot}6H_2O$, $Fe(NO_3)_3{\cdot}6H_2O$, ethanol (EtOH), thioacetamide (TAA), cyclohexane, were A.R. and purchased from Sinopharm Chemical Reagent Co. Ltd. Oleylamine (OAm) was A.R. and purchased from the Acros Organics. Commercial IrO_2 catalyst was purchased from Alfa Aesar Co. Ltd. All reagents were used as received without further purification.

Synthesis of the pyrite porous cubes (PCs): In a typical synthesis of the PC-1, 0.029 g of $Ni(NO_3)_2{\cdot}6H_2O$ (0.1 mmol) was dissolved in 0.1 mL EtOH, then added into 3 mL of OAm and stirred for several minutes in an autoclave. At the meantime, 0.034 g of TAA (0.45 mmol) was dissolved in 1 mL of OAm, and added into the $Ni(NO_3)_2{\cdot}6H_2O$/OAm solution under vigorous stir. The stir was kept for another 5 min. Then the autoclave was sealed and heated at 180°C for 4 hours, after which it was allowed to cool naturally to room temperature. The resulting products were separated by centrifugation and washed with cyclohexane and EtOH several times. The PC-2, PC-3 and PC-4 were prepared in a similar way, by adding 0.005 g (0.013mmol), 0.010 g (0.025mmol) and 0.020 g (0.05mmol) of $Fe(NO_3)_3{\cdot}6H_2O$ together with 0.029 g of $Ni(NO_3)_2{\cdot}6H_2O$ (0.1 mmol) at the beginning, respectively. The compositions determined by the inductively coupled plasma optical emission spectrometry (ICP-OES) of the PC-2, PC-3 and PC-4 were $(Ni_{0.86}Fe_{0.14})S_2$, $(Ni_{0.74}Fe_{0.26})S_2$ and $(Ni_{0.63}Fe_{0.37})S_2$, respectively.

Synthesis of the (oxy)hydroxides: In a typical synthesis of OH-1, 0.29 g of $Ni(NO_3)_2{\cdot}6H_2O$ (1mmol) was dissolved in 15 mL of EtOH in an autoclave, then 3 mL of OAm were added into the solution, followed by adding 15 mL of cyclohexane. Then the autoclave was sealed and heated at 100°C for 4 hours, after which it was allowed to cool naturally to room temperature. The resulting products were separated by centrifugation and washed with cyclohexane and EtOH several times. The OH-2 and OH-3 were prepared in a similar way, by adding 0.06 g (0.15mmol), 0.12 g (0.3 mmol) of $Fe(NO_3)_3{\cdot}6H_2O$ together with 0.29 g of $Ni(NO_3)_2{\cdot}6H_2O$ (1 mmol) at the beginning, respectively. The ratio of Ni to Fe determined by the inductively coupled plasma optical emission spectrometry (ICP-OES) of the OH-2 and OH-3 were 6.9:1 and 3.2:1, respectively.

Electrochemical tests: The electrochemical tests were performed in 1M KOH solutions and conducted with a CHI650D electrochemical workstation using a three electrode configuration. Pt foil served as the counter electrode and Ag/AgCl electrode were used as the reference electrode. RHE potential was calculated as E(RHE) = E(vs Ag/AgCl) + 0.198 + 0.0592*pH. Before electrochemical test, the electrolyte solutions were purged with O_2 for at least 30mins. The working electrodes were prepared in this way: (1) materials (about 5 mg) were first loaded on Ketjin carbon (about 1mg) by dispersing them in cyclohexane, and sonicated for at least 30mins, followed by centrifugation and drying; (2) the carbon loaded materials were dispersed in water/ethanol/Nafion (5%) solutions (volume ratio:0.7:0.25:0.05) and sonicated for at least 30mins to prepare the ink; (3) then 14uL of the ink was dropped on the surface of a rotating disk electrode (0.196cm^2) and allowed to dry naturally. The loading amounts of metal species on the electrodes were determined by the ICP-OES (Results shown in Table S1). The current density provided in this paper was normalized by the geometry area of the electrode (0.196cm^2). The LSV plots were recorded by applying proper potential ranges at the scan speed of 10mV/s and the Tafel plots were recorded at the scan speed of 1mV/s at proper potential ranges. The rotating ring-disk electrode (RRDE) tests were conducted with a CHI 760D electrochemical workstation. Before electrochemical test, the electrolyte solutions (1M KOH) were purged with N_2 for at least 30mins. The collecting efficiency of the RRDE were measured in a solution of 0.1M KOH with 10 mM $K_3Fe(CN)_6$, and the results showed a collection efficiency of N=4.0.

The turnover frequency (TOF) were calculated based on all Ni and Fe amounts on the electrodes, by:

$$TOF = \frac{I(mA)}{4 * 96500 * (\frac{m(mg,Ni)}{58.69} + \frac{m(mg,Fe)}{55.85})}$$

Characterizations: The X-ray diffraction (XRD) patterns were performed on a Bruker D8 ADVANCE X-ray powder diffractometer operated at 40 kV voltage and 40 mA current with Cu Kα radiation (λ = 1.5406 Å). The X-ray photoelectron spectroscopy (XPS) results were achieved by ESCALAB 250Xi (Thermo Fisher). The calibrations were done with respect to carbon C1s. The morphology and size of the products were determined by a Hitachi H-7700 transmission electron microscope (TEM) at 100 kV, a JEOL 2100F HRTEM equipped with energy dispersive X-ray spectroscopy (EDS) at 200 kV, was used to perform high-angle annular-dark-field (HAADF)-STEM and EDS mapping. Inductively coupled plasma optical

emission spectrometry (ICP-OES) was used to determine the composition of the mass loading for electrochemical test. The soft X-ray adsorption spectrum (sXAS) measurements were carried out the at the beamline 4W9B in Beijing Synchrotron Radiation Facility (BSRF). The X-ray was monochromatized by a double-crystal Si (111) monochromator for BSRF. The energy was calibrated using a gold metal foil.

Supporting Fig.s:

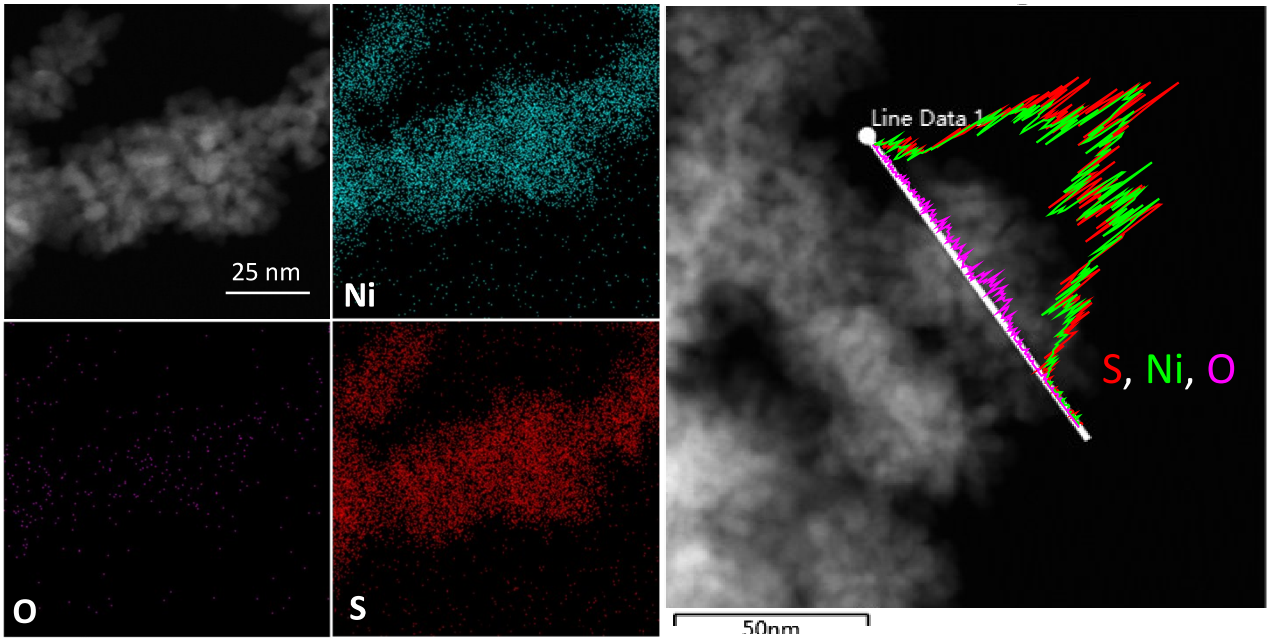

Fig. S1. Element mapping and line scanning profile of the PC-1

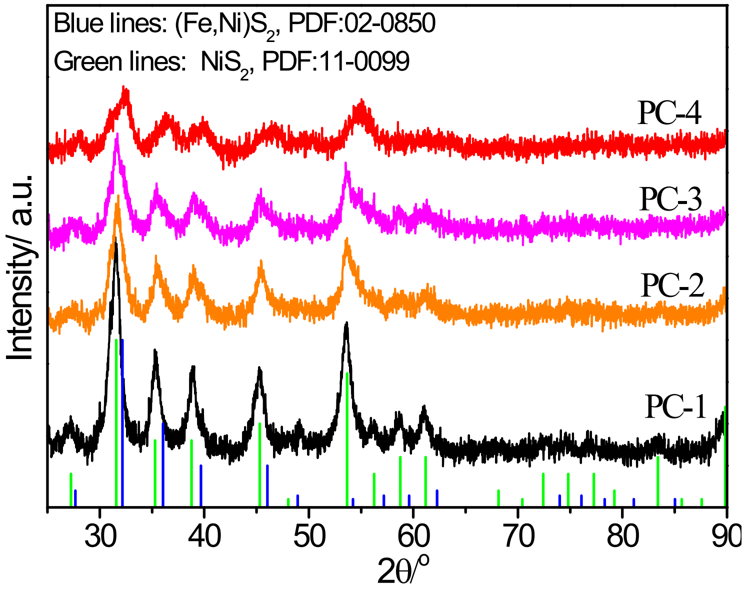

Fig. S2. XRD patterns of the PC structures

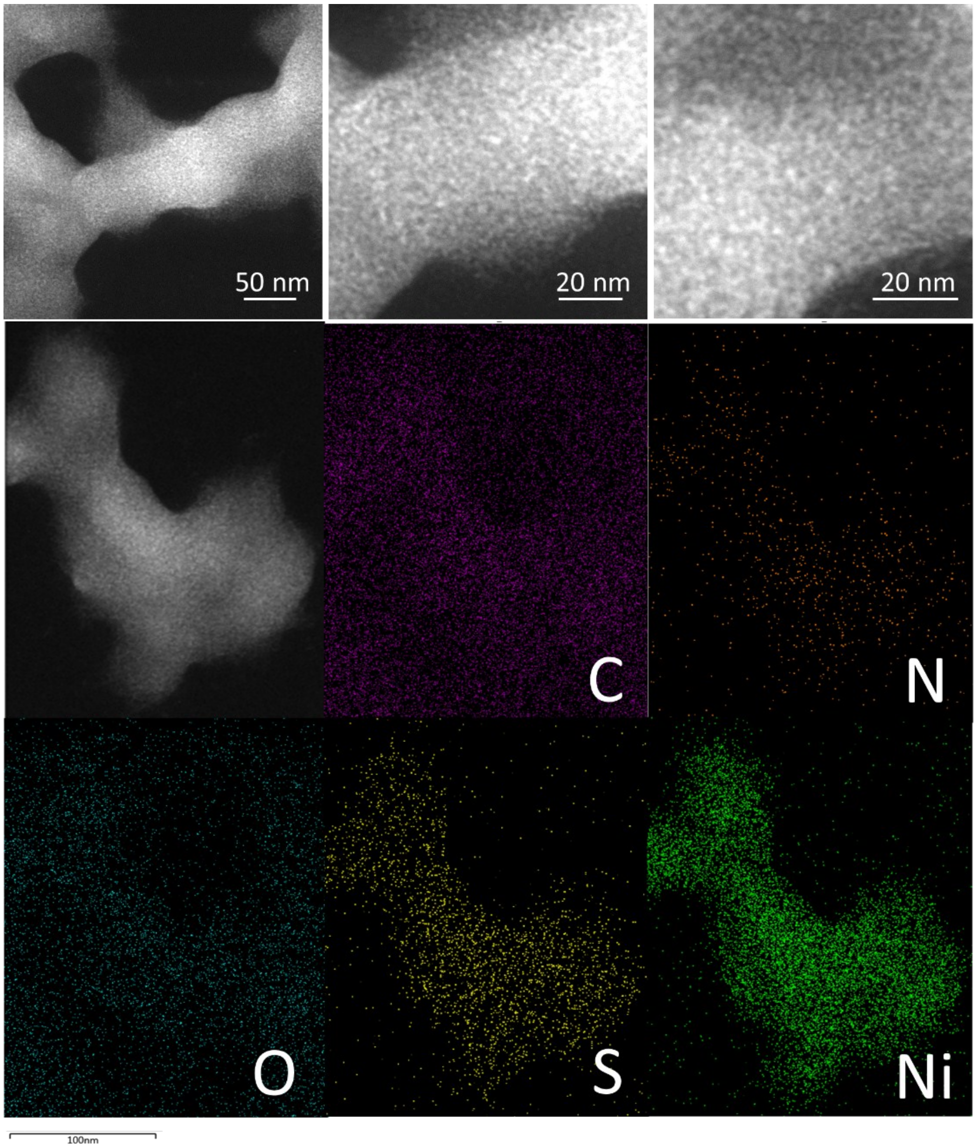

Fig. S3. STEM images and elemental mapping results of the clusters embedded in a gel.

Although the carbon grid for TEM testing contains C and O, the shape of clusters embedded in gel can almost be identified from the signal of C and O in the mapping results. Moreover, the signal of N (results from OAm in the synthesis) can superimpose the gel. Together with the signals of S and Ni, the black precipitates when mixing all precursors can be inferred to be small nickel sulfide clusters of less than 2nm embedded in a gel.

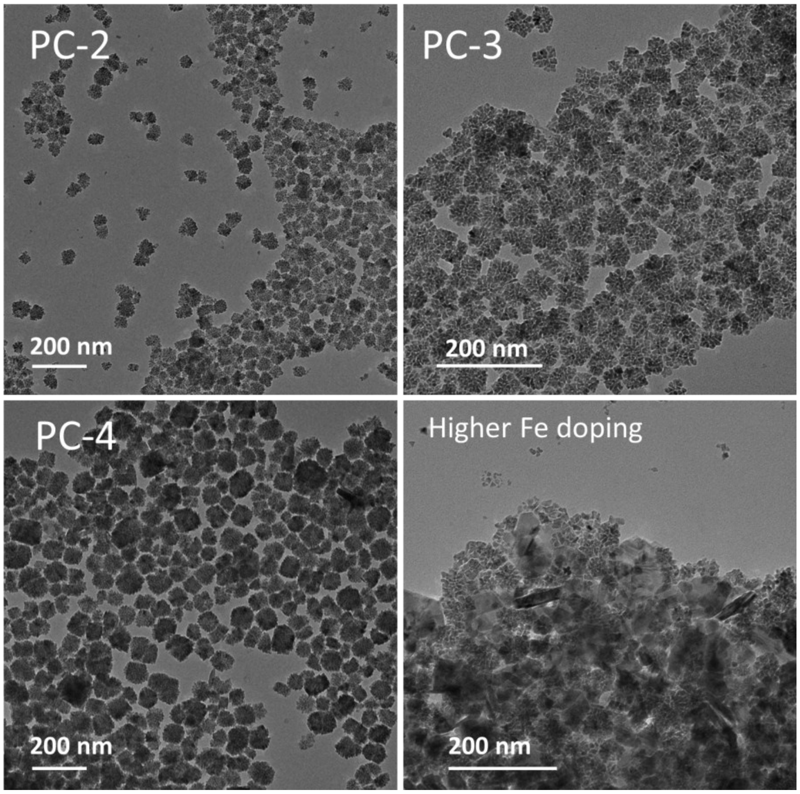

Fig. S4. TEM images of the Fe doped PC structures.

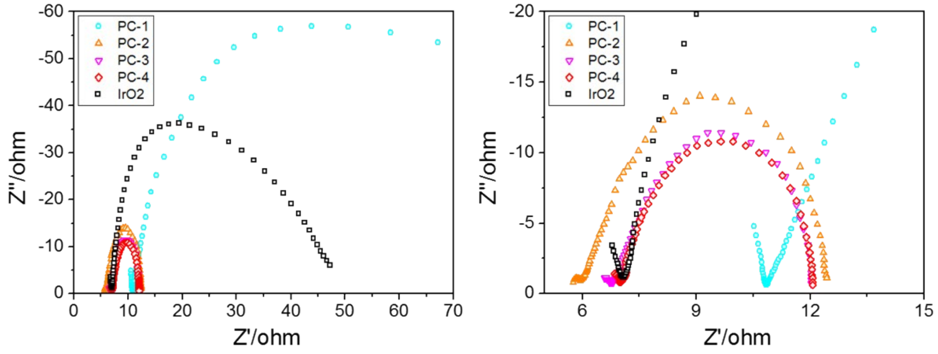

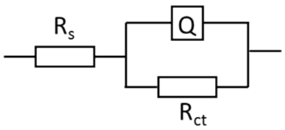

	R_s/Ω	R_{ct}/Ω	$Y_o/(S\cdot s^{-n})$	n
PC-1	11.05	206.7	0.01356	0.861
PC-2	6.019	6.971	0.01814	0.7097
PC-3	6.817	5.608	0.01968	0.7438
PC-4	7.033	5.315	0.01723	0.7724

Fig. S5. Electrochemical impedance spectra. Frequency range: 0.1Hz to 100k Hz. Potential: 1.53V (vs RHE).

The figure and table in the lower panel indicate the Randles model used in this report and the corresponding simulation results (the simulation was achieved using ZSimpWin software). The reason that imaginary impedance increases at high frequency is attributed to the contributions from cable and instrument capacitances and inductances. The R_s is estimated by using the impedance at high frequency where the phase angle is closest to 0 degrees[1].

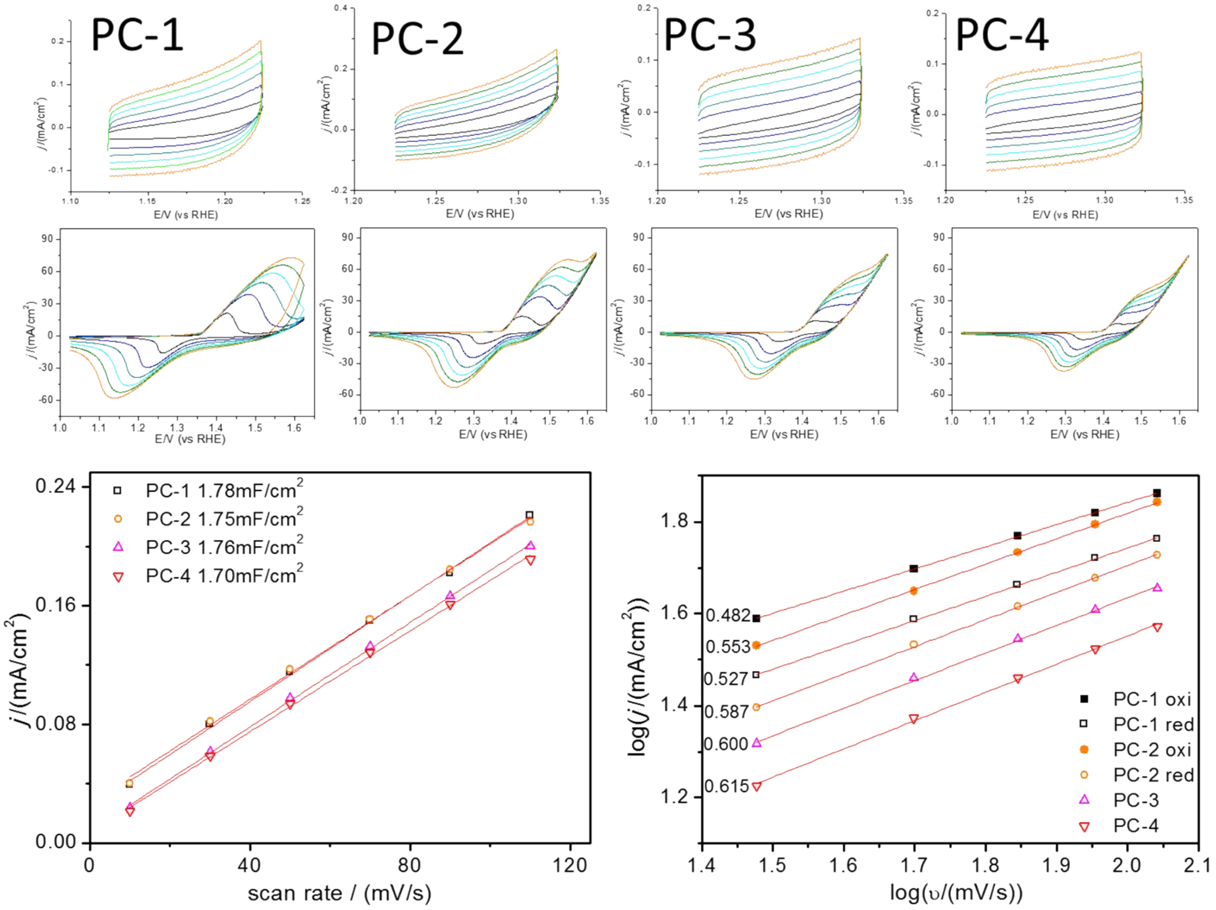

Fig. S6. CV scans at different scanning rate to estimate the ECSA and oxidation mechanism (surface vs bulk).

The upper row shows the CV scans of different materials with different scanning rate (10, 30, 50, 70, 90, 110 mV/s) at a potential range including their open circuit potentials. The ECSA were estimated from the electrochemical double layer capacitance[2] (C_{dl}). The $2C_{dl}$ was estimated by plotting the Δj (j_{anodic} - $j_{cathodic}$) at a given potential against the scan rate (left graph in the lowest row).

The middle row shows the CV scans of different materials with different scanning rate (10, 30, 50, 70, 90, 110 mV/s) at a potential range including their oxidation and reduction peaks. Generally, the oxidation peak current obeys the power law relation with the scan rate[3] (υ): $j = a\upsilon^b$. The value of *b* can be calculated by the slope of the logj verses logυ plot (right graph in the lowest row, slopes are shown in the left). There are two well-defined conditions[4]: (1) If *b* is close to 0.5, the faradaic reactions happed in the bulk; (2) If *b* is close to 1.0, it indicates a surface-reaction limited response. Since the oxidation peak cannot be easily differentiated in the case of the PC-3 and PC-4, we used the reduction peak current here. From the results of the PC-1 and PC-2, the conclusion made based on oxidation peak current and reduction peak current was similar. All of them suggests a faradaic redox process in the bulk.

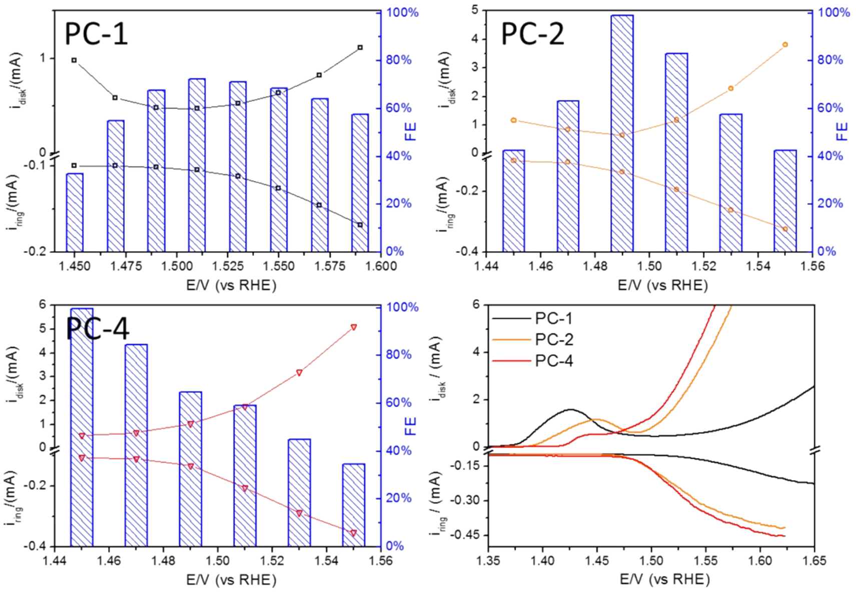

Fig. S7. The Faradaic efficiency (FE) was measured based on the RRDE tests.

$$FE = \frac{n_{app} \times i_r}{i_d \times N}$$

Where n_{app} is the apparent number of electrons, and it was reported to be 2 at a rotating rate of 1600 rpm[2]; i_r and i_d are the measured ring and disk currents, respectively, and N is the collection efficiency (4.0). The decrease of Faradaic efficiency with the increase of potential at the disk electrode was caused by the generation of oxygen bubbles[5]; only dissolved oxygen can be reduced at the surface of the Pt ring electrode. When the current density (about 1 mA/cm^2, 0.3mA in the graphs) at this potential is sufficiently large to generate fully dissolved oxygen without forming oxygen bubbles at the disk electrode. Thus, the calculated FE would decrease as the current increased. The calculated FE of 96% and 98% at 1.49V and 1.45V (of the PC-2 and PC-4, respectively) is representative of their OER FE.

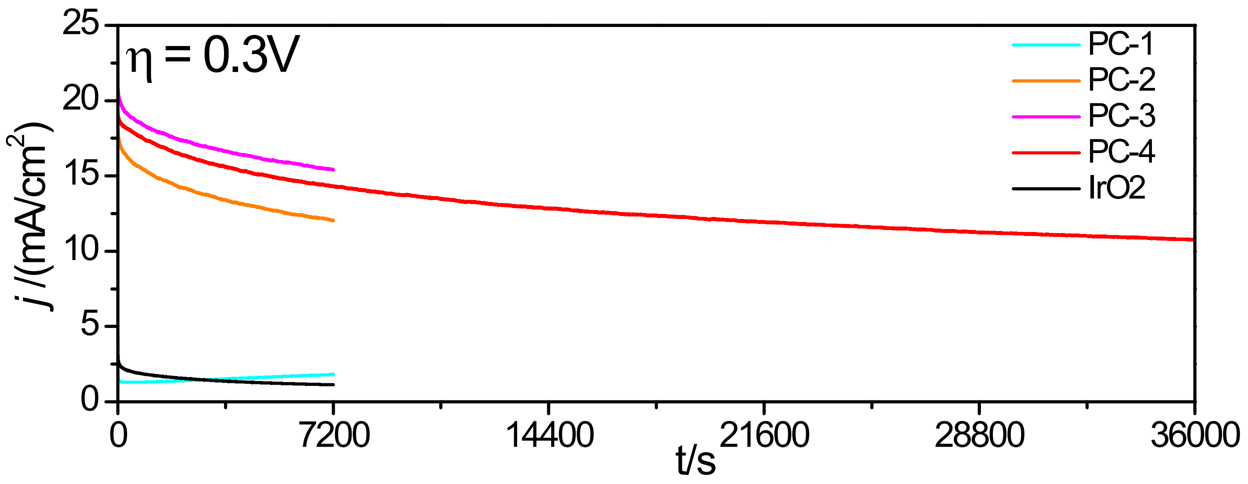

Fig. S8. Chronoamperometry tests.

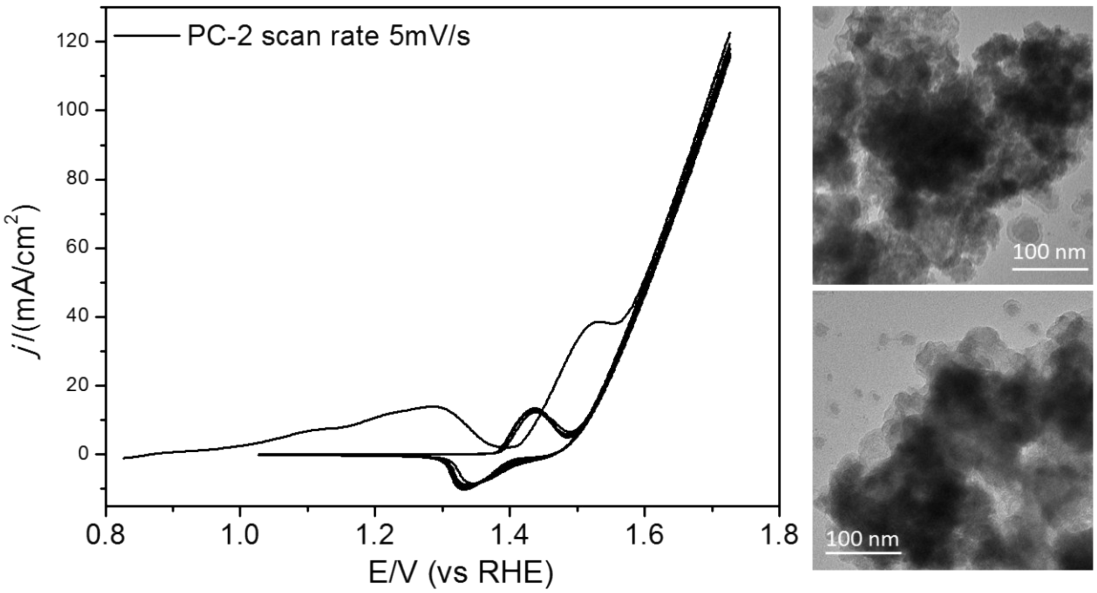

Fig. S9. CV scan at lower scan rate and the corresponding TEM images of PC-2 after the scan.

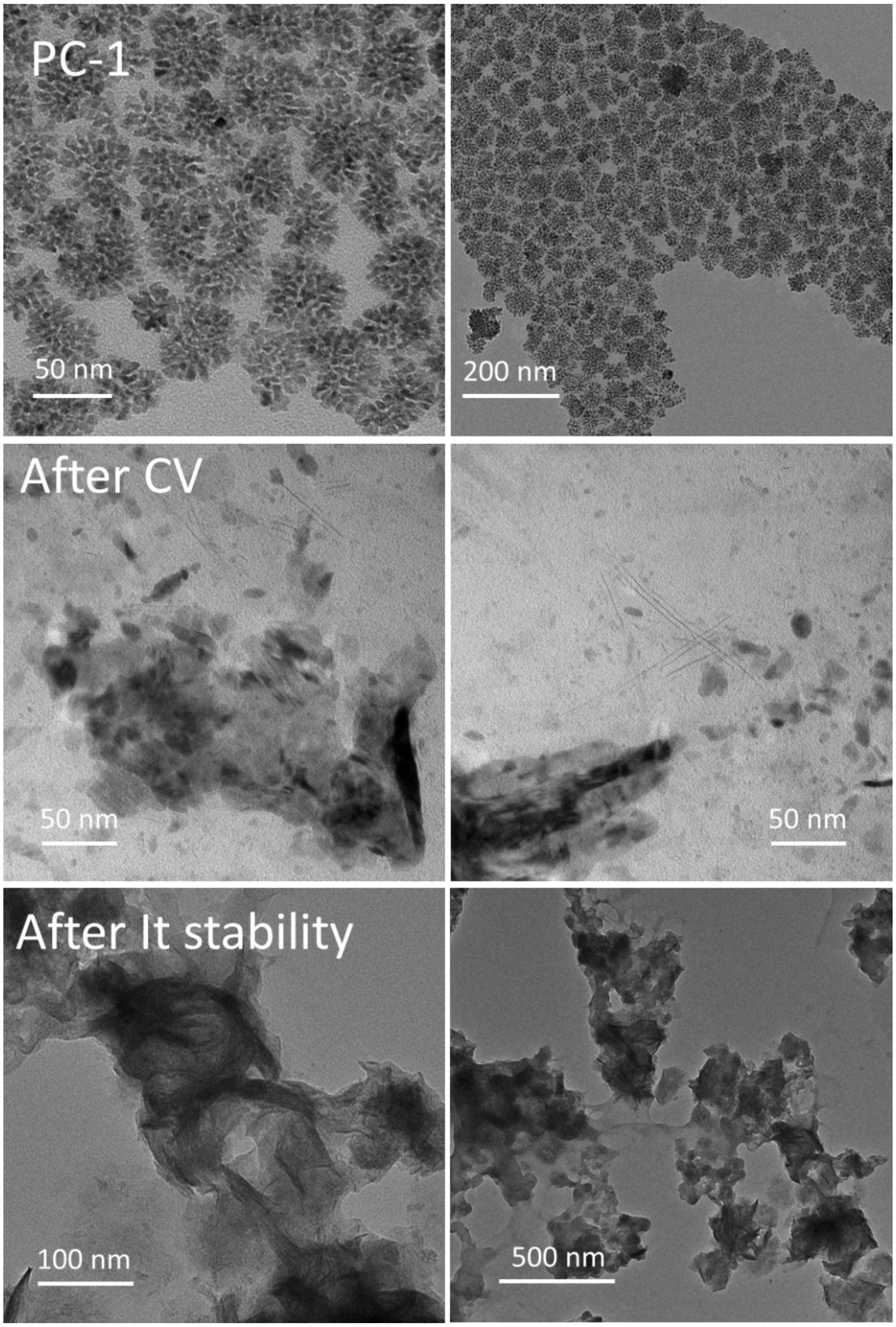

Fig. S10. TEM images of the PC-1 before and after OER tests. (It stability: η=300mV, 1h).

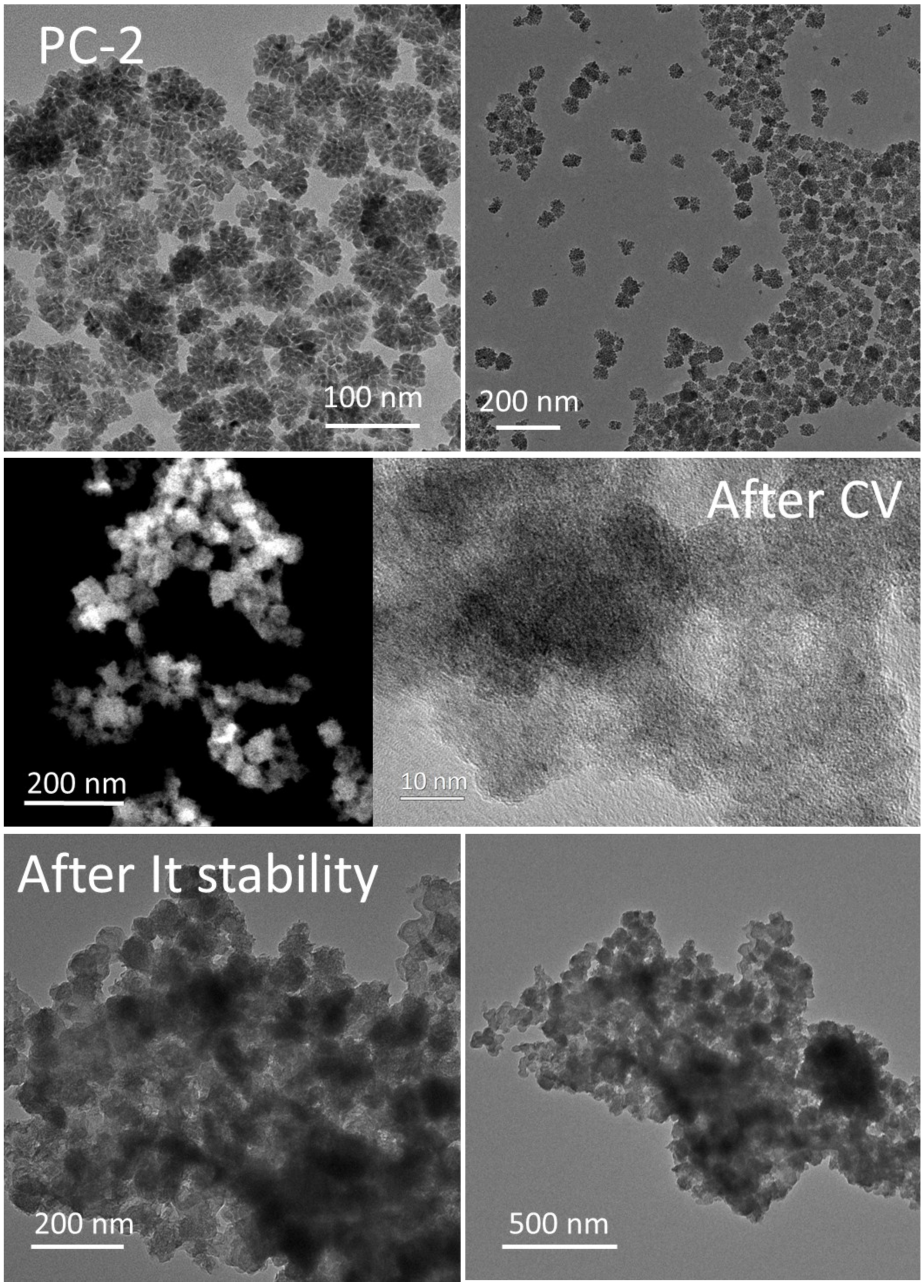

Fig. S11. TEM images of the PC-2 before and after OER tests. (It stability: η=300mV, 1h).

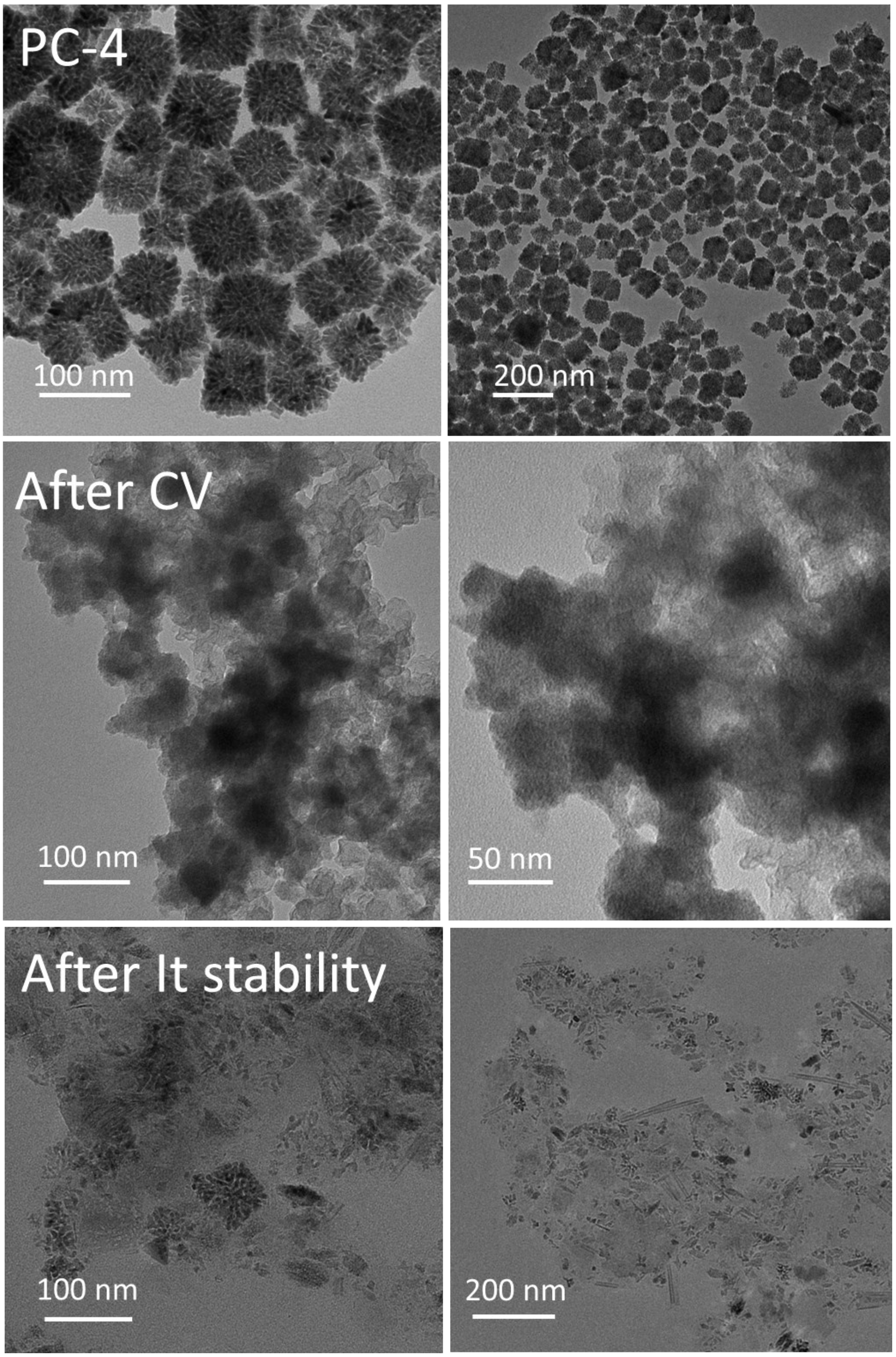

Fig. S12. TEM images of the PC-4 before and after OER tests. (It stability: η=300mV, 1h).

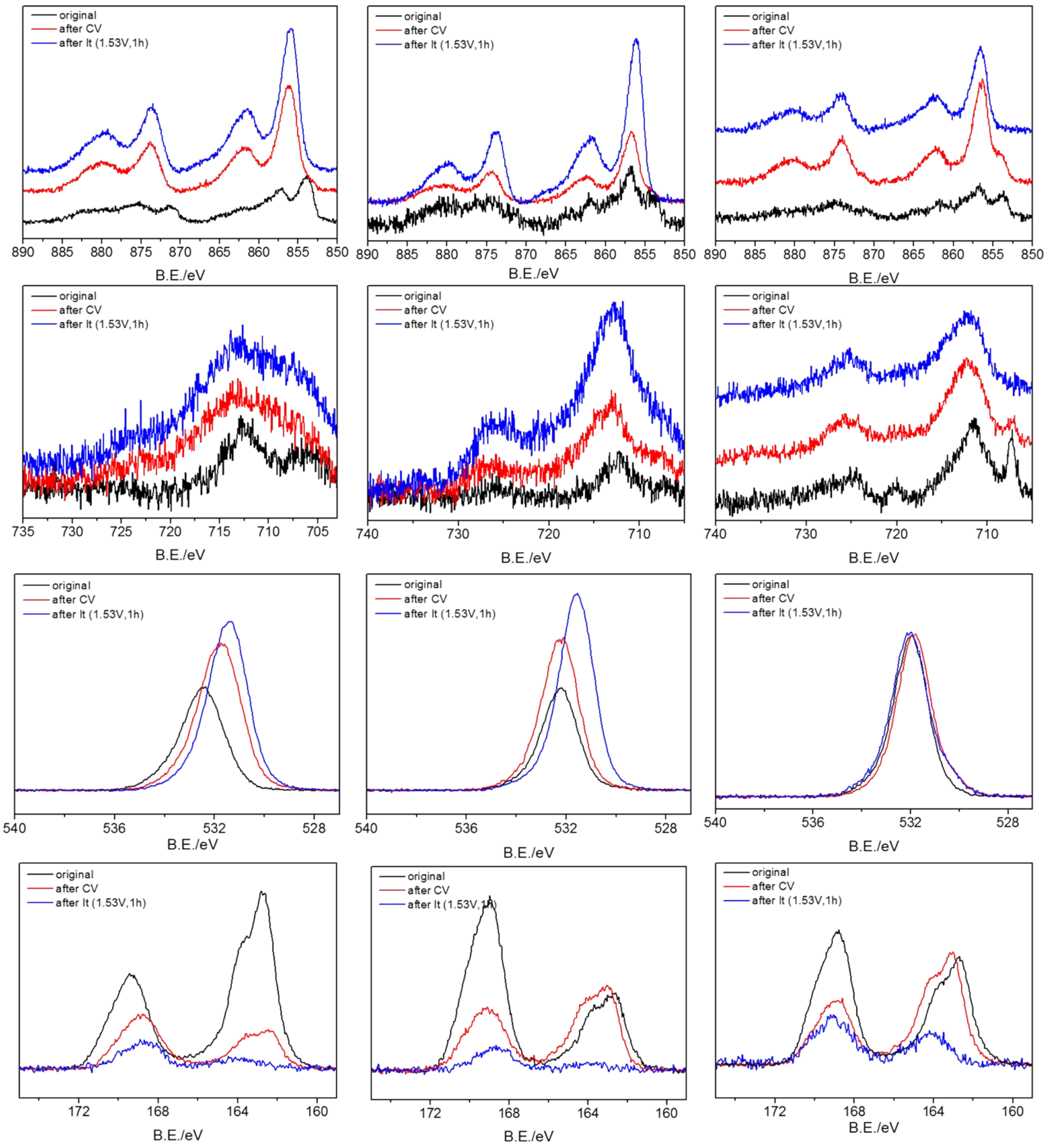

Fig. S13. XPS spectra of the PC materials before and after OER tests.

The spectra of Ni_{2p}, Fe_{2p}, O_{1s} and S_{2s} scans are shown in the first, second, third and fourth row respectively. The analysis of Ni_{2p} and Fe_{2p} scans is showcased in Fig. S12. In the S_{2s} spectra, the peak centered at lower energy arises from S in pyrite[6], the peak centered at higher energy (168-172 eV) arises from the surface absorption of oxygen with sulfur to form S-O bond[7].

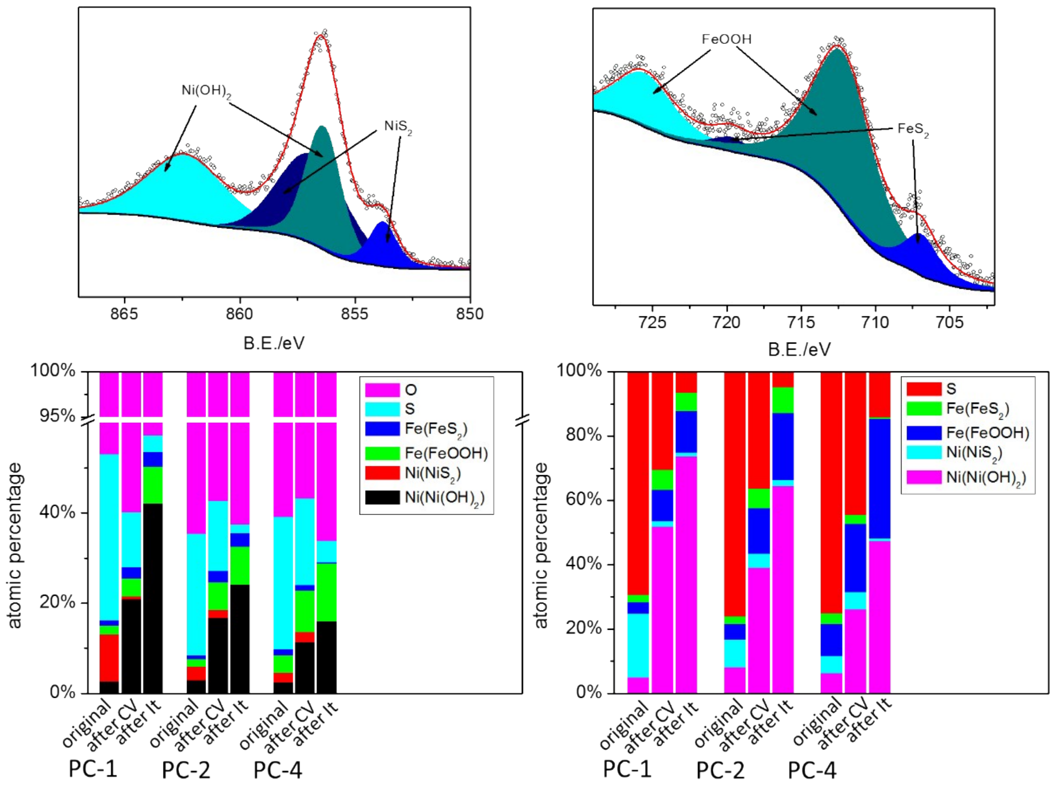

Fig. S14. Composition changes of the PC materials before and after OER tests estimated by the XPS spectra.

The upper row showcases the analyzing of the Ni_{2p}[8] and Fe_{2p}[6, 9] data using the data of the PC-4 after CV scans. All the data were analyzed in the same way, and the results are shown in the lower row (the amount of O is considered in the left and omit in the right). The red color in the right panel indicate the amount of S, and other colors indicate metals, we can infer that the relative amount of remaining S (to total amount of metals) would be higher with higher content of Fe doping from this graph.

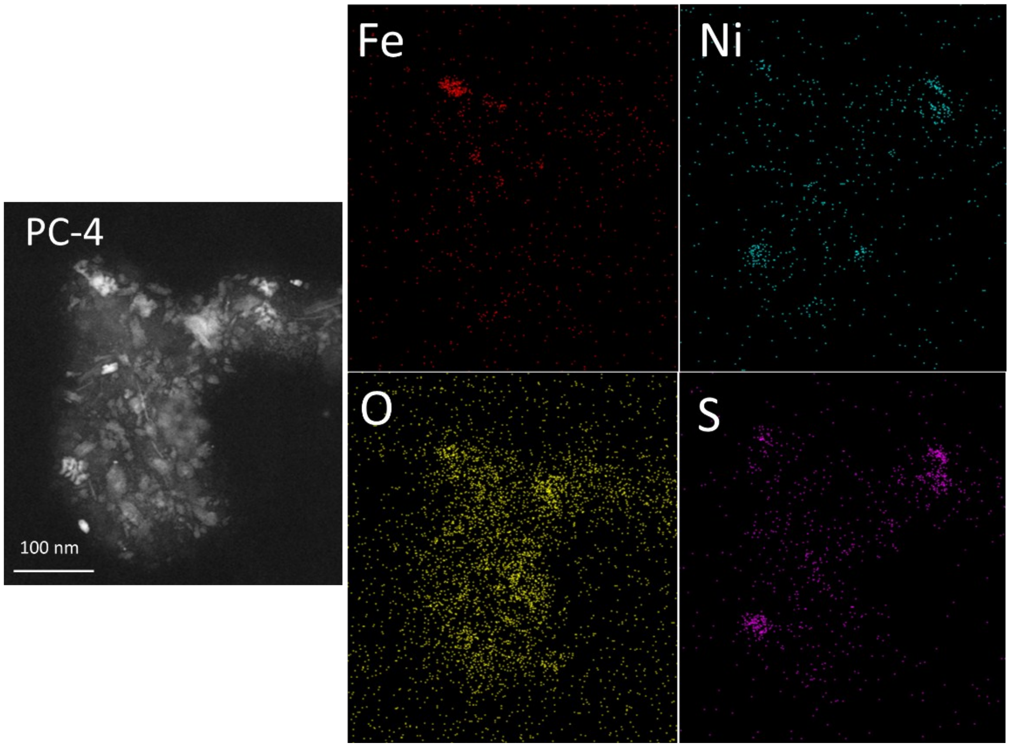

Fig. S15. Element mapping of the PC-4 after It stability (η=300mV, 1h).

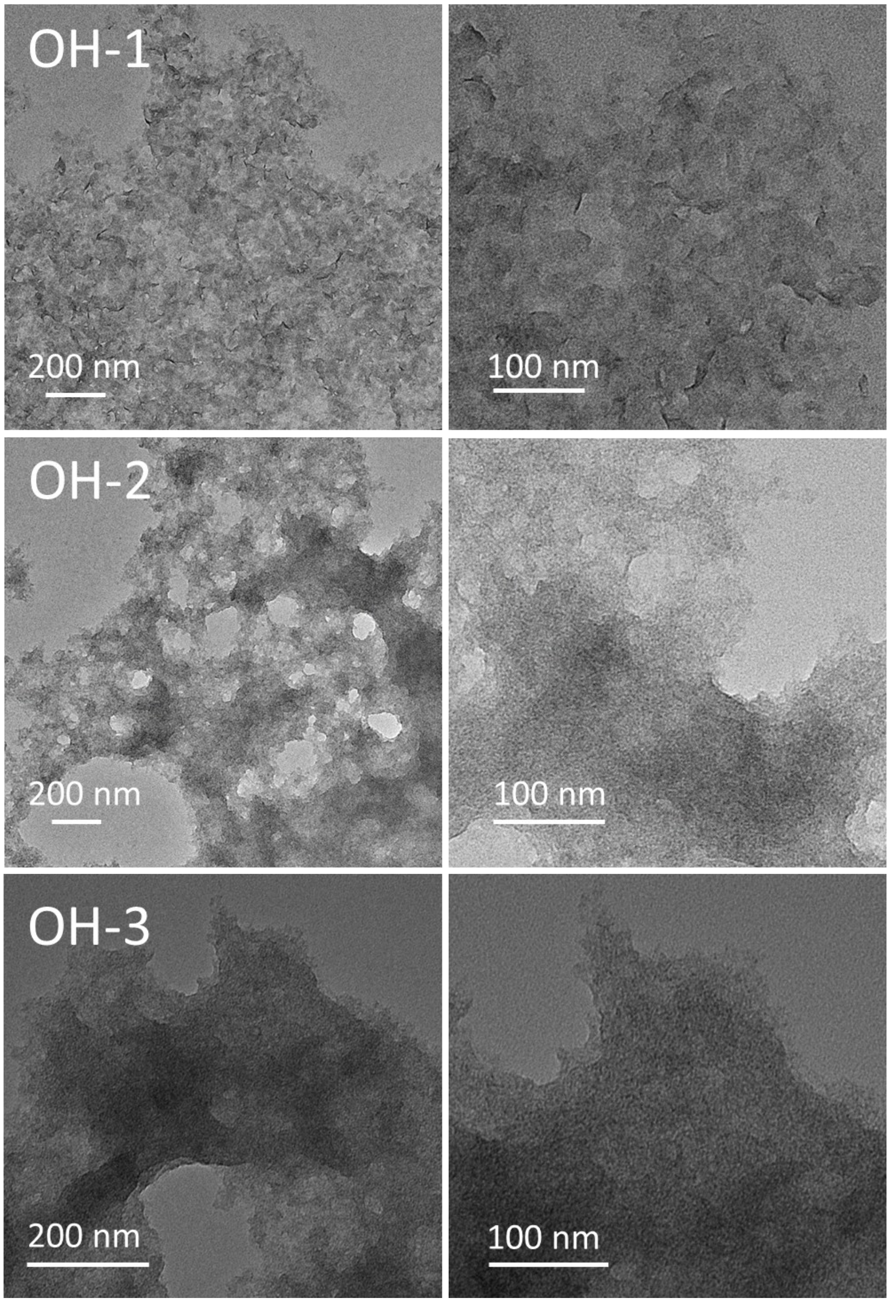

Fig. S16. TEM images of the (oxy)hydroxides.

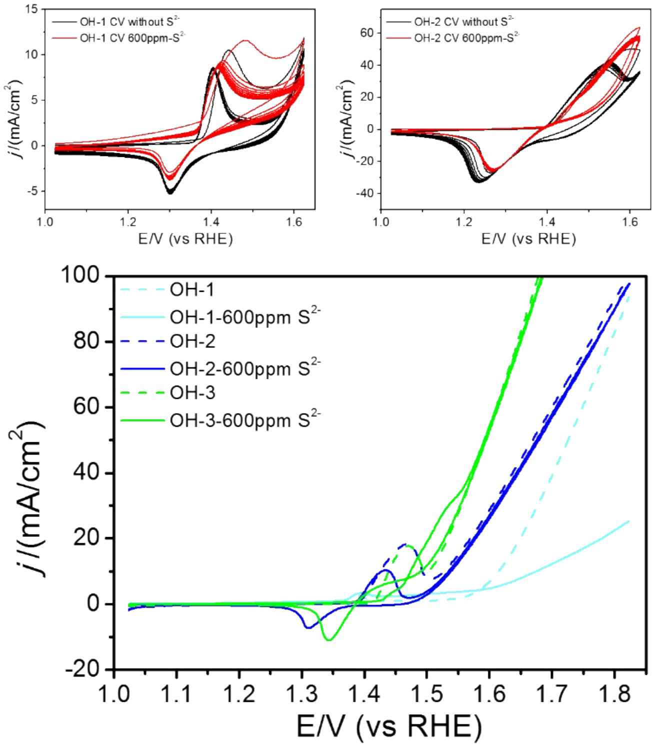

Fig. S17. CV curves of the (oxy)hydroxides with or without 600ppm S^{2-} in the electrolyte.

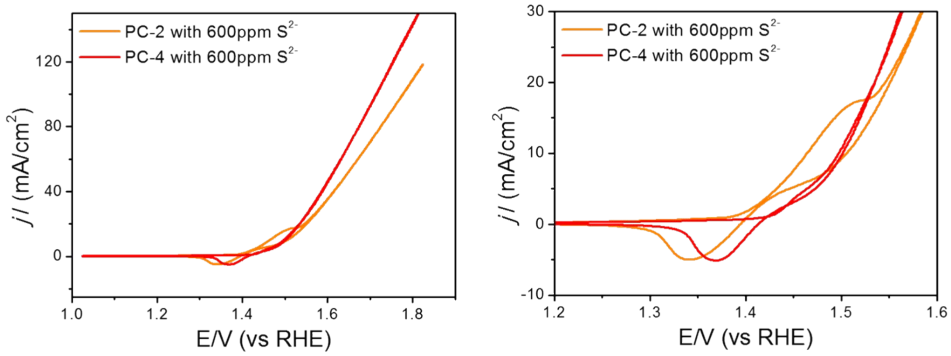

Fig. S18. CV curves of the PC materials with 600ppm S^{2-} in the electrolyte.

Supplementary tables

Table S1. A summary of the OER activity.

Catalyst	Loading [a] (mg/cm^2)	η [b](mV, j=10mA/cm^2)	TOF [c] (s^{-1})	Tafel slope (mV/dec)	Ref [d]
PC-1	0.102<br>0.102<br>~0	373	0.0046	75.6	This work
PC-2	0.109<br>0.0938<br>0.0148	264	0.035	56.2	
PC-3	0.101<br>0.0760<br>0.0251	269	0.034	52.4	
PC-4	0.0996<br>0.0638<br>0.0358	262	0.034	56.4	
OH-1	0.189<br>0.189<br>~0	385	0.0010	56.4	
OH-2	0.150<br>0.132<br>0.0182	297	0.011	59.8	
OH-3	0.167<br>0.129<br>0.0378	271	0.017	50.9	
IrO_2	0.143	330	0.020	44.6	
FeCo LDH	0.21	331	0.0085	85	10
Gelled-FeCo		277	0.043	60	
Gelled-FeCoW		223	0.46	37	
Annealed-FeCoW		301	0.17	70	
CoFe oxide frame-like superstructure	0.1	340		57	11
CoFe oxide nanoframe		350		61	
Holey $NiCo_2Se_4$ sheets	0.285	295	0.016	53	12
Bare NiS_2	0.272	~325		65	13
5% V doped NiS_2		~310		50	
10% V doped NiS_2		290		45	
25% V doped NiS_2		~380		69	
Porous hollow microspheres NiS	0.7±0.2	320		59	14
Ni_3S_2@C/CNS	0.25	298		48	15
NiS_x	0.5	353		56	16
$NiSe_2$ nanosheets		330		80	17
CoS_2 porous cube	0.8	290		67	7
Bulk CoS_2		480		160	
Amorphous nickel (II) borate shelled Ni_3B	0.3	364	0.008	85	18
Activated MnCo oxyphosphide hollow sphere	0.25	370		52	19
MnCo oxyphosphide hollow sphere		420		66	
$NiPS_3$	0.51	437 (20mA/cm^2)		73	20
$Ni_{0.9}Fe_{0.1}PS_3$		329 (20mA/cm^2)		69	

a. the first row is the total metal loading including Ni and Fe, the second and third rows are the loading of Ni and Fe, respectively. (loading in Ref. is the total amount of catalysts)

b. without iR compensation.

c. TOF is calculated based on the total metal loading at η=300mV.

d. The reported data were also acquired on glassy carbon electrodes.

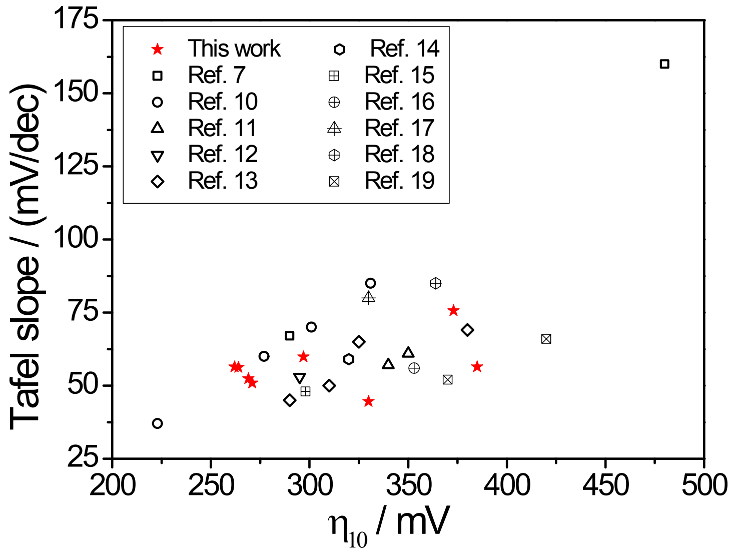

A brief summary of the OER activities according to their overpotential required to reach a current density of $10mA/cm^2$ and their Tafel slopes. The dots at the left bottom indicates better performance.

Table S2. The contents of high valence metals.

		Ni	Fe
OH-1		0.25	
OH-2		0.21	7.0
OH-3		0.26	10.6
PC-1	Pristine	0.32	
	After CV scan	0.29	
PC-2	Pristine	0.32	2.0
	After CV scan	0.27	6.13
PC-3	Pristine	//	2.5
	After CV scan		5.32

The values indicate the ratio of high valence to low valence, estimated by the deconvolution of the spectra (examples are shown below). It's worth to mention that this method can only qualitative tell the ratio of high valence, but cannot explicitly determine the valence of metals and their ratios. The qualitative results can suggest that: In the case of the PC-2 materials, the spectra would become more similar with that of the (oxy)hydroxide after CV scans, with similar ratio of high valence metals. However, the PC-4 materials showed different features, with lower ratio of high valence metals. But the PC-4 pre-catalysts indeed showed higher activity.

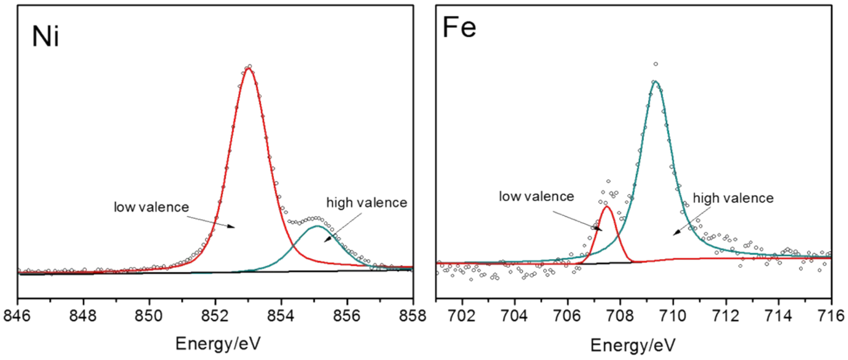

Reference

1. M. B. Stevens, L. J. Enman, A. S. Batchellor, M. R. Cosby, A. E. Vise, C. D. M. Trang and S. W. Boettcher, *Chem. Mater.*, 2017, **29**, 120-140.
2. C. C. L. McCrory, S. Jung, J. C. Peters and T. F. Jaramillo, *J. Am. Chem. Soc.*, 2013, **135**, 16977-16987.
3. H.-Y. Wang, S.-F. Hung, Y.-Y. Hsu, L. Zhang, J. Miao, T.-S. Chan, Q. Xiong and B. Liu, *Journal of Physical Chemistry Letters*, 2016, **7**, 4847-4853.
4. T. Brezesinski, J. Wang, J. Polleux, B. Dunn and S. H. Tolbert, *J. Am. Chem. Soc.*, 2009, **131**, 1802-1809.
5. X. Yu, M. Zhang, W. Yuan and G. Shi, *J. Mater. Chem. A*, 2015, **3**, 6921-6928.
6. K. Sasaki, M. Tsunekawa, T. Ohtsuka and H. Konno, *Geochim. Cosmochim. Acta*, 1995, **59**, 3155-3158.
7. P. Cai, J. Huang, J. Chen and Z. Wen, *Angew. Chem., Int. Ed.*, 2017, **56**, 4858-4861.
8. H. Vanderheide, R. Hemmel and C. F. Vanbruggen, *J. Solid State Chem.*, 1980, **33**, 17-25.
9. N. S. McIntyre and D. G. Zetaruk, *Anal. Chem.*, 1977, **49**, 1521-1529.
10. B. Zhang, X. Zheng, O. Voznyy, R. Comin, M. Bajdich, M. Garcia-Melchor, L. Han, J. Xu, M. Liu, L. Zheng, F. P. G. de Arquer, C. T. Dinh, F. Fan, M. Yuan, E. Yassitepe, N. Chen, T. Regier, P. Liu, Y. Li, P. De Luna, A. Janmohamed, H. L. Xin, H. Yang, A. Vojvodic and E. H. Sargent, *Science*, 2016, **352**, 333-337.
11. J. Nai, B. Y. Guan, L. Yu and X. W. Lou, *Sci. Adv.*, 2017, **3**, e1700732.
12. Z. Fang, L. Peng, H. Lv, Y. Zhu, C. Yan, S. Wang, P. Kalyani, X. Wu and G. Yu, *ACS Nano*, 2017, **11**, 9550-9557.
13. H. Liu, Q. He, H. Jiang, Y. Lin, Y. Zhang, M. Habib, S. Chen and L. Song, *ACS Nano*, 2017, **11**, 11574-11583.
14. P. Luo, H. Zhang, L. Liu, Y. Zhang, J. Deng, C. Xu, N. Hu and Y. Wang, *ACS. Appl. Mater. Inter.*, 2017, **9**, 2500-2508.
15. M. Al-Mamun, H. Yin, P. Liu, X. Su, H. Zhang, H. Yang, D. Wang, Z. Tang, Y. Wang and H. Zhao, *Nano Res.*, 2017, **10**, 3522-3533.
16. H. Li, Y. Shao, Y. Su, Y. Gao and X. Wang, *Chem. Mater.*, 2016, **28**, 1155-1164.
17. S. Chen, Z. Kang, X. Hu, X. Zhang, H. Wang, J. Xie, X. Zheng, W. Yan, B. Pan and Y. Xie, *Adv. Mater.*, 2017, **29**, 1701687.
18. W.-J. Jiang, S. Niu, T. Tang, Q.-H. Zhang, X.-Z. Liu, Y. Zhang, Y.-Y. Chen, J.-H. Li, L. Gu, L.-J. Wan and J.-S. Hu, *Angew. Chem., Int. Ed.*, 2017, **56**, 6572-6577.
19. B. Y. Guan, L. Yu and X. W. Lou, *Angew. Chem., Int. Ed.*, 2017, **56**, 2386-2389.
20. B. Song, K. Li, Y. Yin, T. Wu, L. Dang, M. Cabán-Acevedo, J. Han, T. Gao, X. Wang, Z. Zhang, J. R. Schmidt, P. Xu and S. Jin, *ACS Catal.*, 2017, **7**, 8549-8557.
